# Privacy Preservation in Smart Meters: Current Status, Challenges and Future Directions

**DOI:** 10.3390/s23073697

**Published:** 2023-04-03

**Authors:** Jonathan Kua, Mohammad Belayet Hossain, Iynkaran Natgunanathan, Yong Xiang

**Affiliations:** School of Information Technology, Deakin University, Geelong, VIC 3220, Australia

**Keywords:** smart grids, smart meters, privacy preservation, adversaries, renewable energy

## Abstract

Recent years have seen the rapid development of technologies in Smart Grids (SGs) to enhance electricity networks with digital and data communication technologies. SGs can proactively detect, react, and respond to dynamic changes in the network. SGs can also enhance the efficiency and reliability of electricity supplies and promote the integration of renewable energy sources. Smart Meters (SMs) are often seen as the first step to a successful implementation of SGs. While SMs enable Utility Providers and consumers to obtain near real-time information of energy consumption, they can also be exploited to infer sensitive consumer data. Therefore, privacy preservation in SMs is paramount in ensuring the widespread and successful deployment of SGs. In this paper, we present a comprehensive survey of the state-of-the-art SM privacy-preserving techniques published in the literature over the past decade. We categorize these techniques based on the attack types and their objectives. We aim to offer a unique perspective in this survey article through the lens of privacy preservation, cross-cutting the wide range of techniques presented in the literature. We conclude by identifying the challenges and highlighting key future research directions in the field.

## 1. Introduction

In recent years, we have witnessed the rapid development of technologies to support the deployment of Smart Grids (SGs) at scale. SG is a modernized form of power grid that uses advanced communication technologies and information control to achieve two-way information and electricity exchange between consumers and Utility Providers (UPs). SGs aim to seamlessly integrate various technologies to enable consumers and UPs to perform near real-time monitoring of energy consumption, transmission, and generation. This requires the collection of a large amount of data, leading to many data management challenges, one of the most important being the preservation of consumer privacy [1,2,3,4].

Smart Meters (SM) are one of the core SG elements and are often seen as the first step towards the successful implementation of SGs. A compromise in SMs will have serious consequences across the entire SG. SMs enable two-way communication between consumers and the UPs. They can measure and report electricity consumption data in near real-time. An SM enables the UP to optimize and control the distribution and supply of electricity. It also helps to balance the load through the Demand Response (DR) and provides new services for consumers.

SMs can also be used to report the quality of power delivery and detect power outages. High-resolution SM data can be used for dynamic pricing, fraud detection, and demand forecasting in SGs [5]. However, this can lead to a compromise in consumer privacy if these data are not managed properly. For example, an external party can identify individual appliance usage by using Non-Intrusive Load Monitoring (NILM) to analyse SM data.

Sensitive and personal information, such as appliances types/brands, consumer household sizes, age groups, and daily routines, can also be inferred. This information can be leveraged by various companies for targeted advertisements, and more seriously, be exploited by cyber-attackers and adversaries. SM privacy remains one of the most significant challenges that hinders the successful roll-out of SGs in many countries [6].

There is a rich amount of literature that presents a wide variety of techniques to enhance the performance and usability of SMs in SGs. A recent notable survey paper [7] comprehensively reviewed smart meter data analytics from the application perspective. The works of [8,9,10,11,12,13] represent key papers that reviewed key privacy-preserving techniques in recent years.

The work [8] reviewed SM privacy based on data aggregation schemes and discussed challenges related to signal processing, secure cryptographic protocols, and hardware limitations. In [9], a niche area of SM privacy, i.e., Homographic Encryption (HE)-based approaches are reviewed. The work in [10] reviewed, discussed and analysed different SM privacy preservation approaches and then identified their weaknesses and strengths across four categories (information privacy, personal privacy, organization privacy, and intellectual privacy).

The papers in [11,12] focused on the more established aspects of SM privacy without considering newer features, such as value-added services (VAS), SM data alteration approaches and renewable energy sources (RES). The regulations around SM data collection and management are reviewed in [13], and some recent papers [14,15,16] investigated the merits and demerits of various data protection and privacy preservation schemes in SG communications.

Table 1 summarizes the comparison of the notable survey papers in the area of smart meter privacy preservation, and highlights the contributions of our survey paper.

Our intention is to complement the aforementioned papers by writing a dedicated review that expands on and fully addresses the multi-dimensional aspects of privacy preservation in the context of SMs and SGs. In this paper, we review SM privacy-preserving techniques presented in the literature over the past decade. We categorize SM privacy techniques according to the threats from “weak” and “strong” adversaries (as defined in Section 2.4). We cover key aspects of SM privacy preservation including the use of RES, consumer billing, VAS, operational procedures, and cost-friendly techniques. Furthermore, this paper also identify the limitations and provides recommendations for future research and developments. In summary, our key contributions are as follows:

- Provide a detailed taxonomy that captures the current developments and approaches for preserving SM privacy.
- Categorize and comprehensively review state-of-the-art privacy-preserving techniques for SM-based adversarial attack types and objectives.
- Identify key challenges and articulate key future research directions for further developments in the field of SM privacy preservation.

The rest of the paper is organized as follows. Section 2 presents background information and describes SM privacy characteristics/threats. Section 3 and Section 4 review the privacy-preservation techniques against both weak and strong adversaries. Section 5 discusses the challenges and presents future research directions. Section 6 concludes the paper.

## 2. The Smart Grid Infrastructure and the Role of Smart Meters

**Figure 1:**
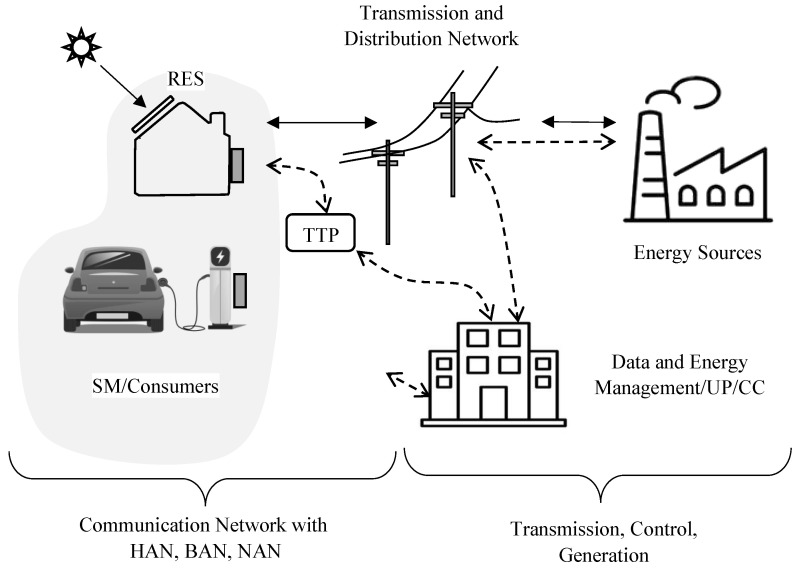
A typical Smart Grid infrastructure (based on [11]). The solid lines denote flow of power, and the dashed lines denote the information flow.

In this section, we present background information on the various entities that make up the SG infrastructure and role of SMs. Figure 1 illustrates the different entities that make up a typical SG infrastructure (based on [11]). The following sections briefly introduce these entities.

### 2.1. Energy Sources, Utility Provider, and Trusted Third Party

Energy generated by sources, such as hydroelectric energy, nuclear energy, and renewable energy, are typically purchased by a Utility Provider (UP) before selling/supplying them to customers via the transmission and distribution network. UP is also known as the Control Center (CC), which controls the smart distribution systems. The UP/CC controls the buying/selling of energy. For example, Figure 1 illustrates how UP/CC controls signals for load balancing, dynamic pricing, and DR program by observing the energy demand. The UP is also responsible for data and energy management in the SG.

Trusted Third Party (TTP) is an entity that facilitates the interactions between two parties (e.g., consumers, UPs, and energy suppliers) and is trusted by both parties. To preserve the privacy of an SG, the TTP reviews all critical transactions between all the parties involved. Generally, a TTP is placed between consumers and the UPs as illustrated in Figure 1.

### 2.2. Smart Meters

SMs represents a critical input point of the smart distribution system. As illustrated in Figure 1, a SM typically resides in the house. It measures the consumer electricity usage with different time granularities and reports the usage to the UP. Information collected by SMs is used by different actors, e.g., the UP uses SM data to predict future energy demand, the electricity market, and the operation of transmission system management. The following summarizes the role of SMs.

- Consumer billing: Information collected by the SMs are used to calculate the energy usage of the consumer for dynamic pricing. Hence, such measurements should be as accurate as possible. The higher the measurement resolution, the more precise the billing of consumer energy usage.
- Accurate SG operations: The UP can predict future energy demand using SM data and can reduce the cost by minimizing the waste of energy. This also improves the efficiency and reliability of the SG. Accurate SM data helps with the state estimation process, load balancing, increases the reliability of power supply and control signals.
- Integration of Renewable Energy Sources (RES): The amount of renewable energy generation is approximated by using SM data. Therefore, the UP can identify the RES required to fulfil the demand of the consumer during peak periods. By analysing SM readings, the consumer can select the type of RES to be installed in their houses. Consumers can reduce the cost by shifting the usage of more appliances in the off-peak periods compared to the peak periods.
- Value Added Services (VAS): The introduction of VAS has assisted in accelerating the electricity market. Customers, third-party service providers, and operators are able to use SM data to provide different VAS, such as management and diagnostics of different electrical appliances.
- Energy-selling-back: By using a smart metering system, the consumer can sell energy back to the grid. Most houses now use RES with solar panels and/or wind turbines.

### 2.3. Renewable Energy Sources in Smart Homes

**Figure 2:**
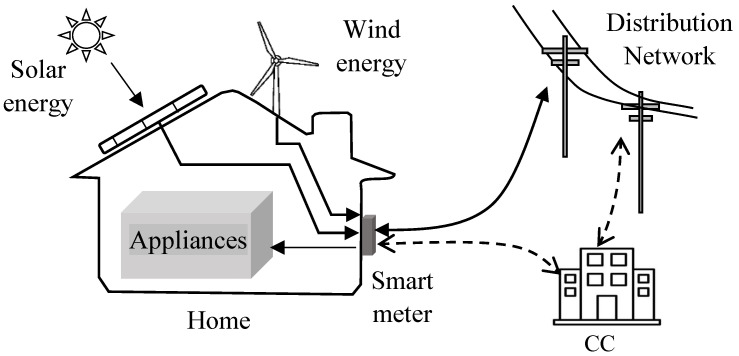
Renewable Energy Source (RES)-based smart metering system [20]. The solid lines denote flow of power, and the dashed lines denote the information flow.

Due to the cost increase of fossil fuel-based energy, many consumers are now interested in integrating RES, such as solar panels and small wind turbines [17]. Due to their dynamic capabilities and heterogeneous nature, the integration of RES with SG is currently undergoing active research [18]. The integration of household RES also helps with reducing the carbon footprint. The consumer can use RES to reduce the cost of electricity. It is predicted that, in the coming future, every house will be equipped with more than one RES [19]. An RES generates energy only for specific hours depending on the weather. Hence, they are used in combination with the main energy supplied from the SG [18]. High-resolution SM data can help the UP to provide information regarding the usage of RES by the consumers. Based on this information, the UP can increase the price of electricity during hours when the RES is not available to generate energy [20]. Figure 2 presents an overview of a RES-based smart metering system.

### 2.4. Smart Meters Privacy

**Figure 3:**
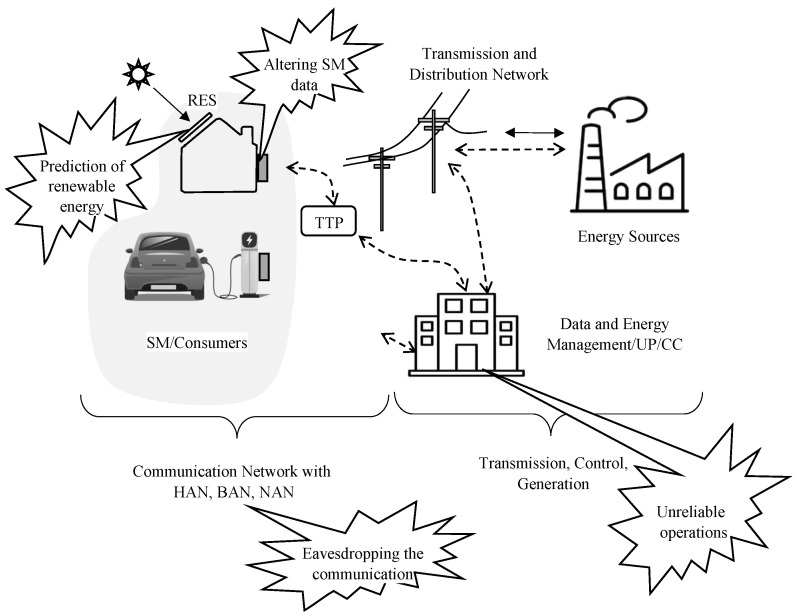
Threat model of SM privacy in SGs. The solid lines denote flow of power, and the dashed lines denote the information flow.

In a SG infrastructure, consumers’ privacy can be breached at any point—starting from the SM in the house, to the communication line, third parties, and the UP itself—posing different threats to SM privacy in SG networks. Figure 3 presents the threat model that we use in this paper.

#### 2.4.1. Weak Adversaries

Weak adversaries have less information on the SM readings. For example, a weak adversary can be an eavesdropper who intercepts the communication between the UP and the SM for a short period. Data alteration methods (such as cryptography, encryption, and the addition of noise) can be used to preserve privacy against weak adversaries. To preserve privacy against a weak adversary, data masking can be performed in the SM or by any trusted authorities. Therefore, anonymity and pseudonymity are very important in hiding the load signature of a consumer. For further improved privacy of SMs against a weak adversary, unobservability and unlinkability should also be considered.

#### 2.4.2. Strong Adversaries

Strong adversaries refer to those who have full access to the SM data reported to the UP. For example, a strong adversary can be one who gains access to the resources of UP or the UP itself. The UP has access to the SM of the consumer and full control over the supply system. Demand-side energy management is used for privacy against the strong adversary. To preserve privacy against a strong adversary, data masking is performed inside the house. Therefore, for hiding the undetectability characteristic of privacy, it is sufficient to consider hiding the load signature of the appliances used in the house.

### 2.5. Threats to Smart Meters Privacy

#### 2.5.1. Predicting Household Renewable Energy Sources

RES (such as solar panels and wind turbines) used by consumers are installed outside of the house as illustrated in Figure 3. Consequently, the UP has direct access to the RES [21]. Therefore, the UP can accurately estimate the RES energy production within each Time Slots (TS) [20] by using information, such as weather forecasts. The demand of the house is usually lower than the normal when the consumers sell renewable energy back to the grid. This information can compromise the privacy of SMs.

#### 2.5.2. Altering Smart Meter Data

SMs are usually installed in a location that is easily accessible and left unprotected most of the time, making them the most exposed device in the SG network. Hence, adversaries can alter SM data (as in illustrated Figure 3) and compromise consumers’ privacy. Energy theft can also be performed by altering the SM data. As a result, SM can be an entry point for physical and side-channel attacks. The software located inside the SM is also vulnerable. SMs are considered to be fully-trusted as they are equipped with a trusted platform module that is used for performing cryptographic primitive by using and storing cryptographic keys [22]. Once the SM is compromised, an attacker can gain access to the keys and data. Large-scale real-time privacy invasion can occur when a large number of SMs are exploited simultaneously.

#### 2.5.3. Eavesdropping in Communication Networks

Secure communication in SG networks is vital in preserving SM privacy. Cryptography-based approaches are adopted to achieve secure communication, and they are being implemented at many points, ranging from the SM to the UP. However, the consumption report of the SM, which is sent via the communication line (or eavesdropping as described in Figure 3) can divulge information that compromises the privacy of customers [23]. Different cyberattacks can also be launched on the exchange messages [24]. Therefore, robust security measures need to be adopted to protect the communication channels.

#### 2.5.4. Unreliable Smart Grid Operations

SG operations should be conducted in such a way that adversary cannot steal the data. The UP collects all the data from the SM. The privacy of the consumers or SMs depends on the reliable operation and accurate control of the SG [25]. Attacks on different devices within the SG operation centre (e.g., internal attacks by a disgruntled employee) can severely compromise SM privacy.

**Figure 4:**
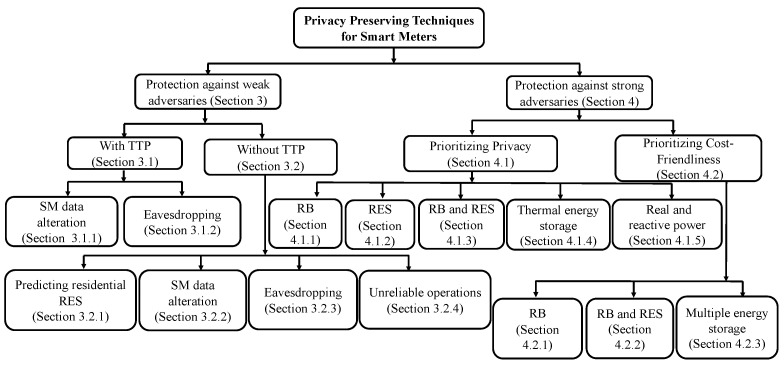
Taxonomy of smart meter privacy-preservation approaches and techniques surveyed and reviewed in this paper.

After considering the potential privacy threats to SMs in SG networks, we will now discuss various state-of-the-art privacy-preservation techniques. SM privacy can be broadly classified into two categories: (i) privacy preservation against *weak adversaries* and (ii) privacy preservation against *strong adversaries*. As defined in Section 2.4, we consider “weak adversaries” as third parties or any intruders who can occasionally eavesdrop on the communication. “Strong adversaries” refers to entities who have access to all the information available to UP. Figure 4 presents the taxonomy of our survey.

### 2.6. Performance Metrics in Smart Meters

#### 2.6.1. Privacy-Preservation Metrics

Privacy-preservation metrics measure the SM’s privacy preservation ability. In demand-side energy management schemes with energy storage, the output of the SM is made different (constant line, rectangular, or other shapes) compared to the real energy demand of a consumer. Most of the models compare the output of SM readings with actual energy consumption to measure their privacy preservation ability. However, not all privacy-preservation metrics are equally suitable for privacy measurements. Below, we describe some commonly used metrics.

*Mutual Information (MI):* MI [26] is a popular metric for measuring theoretic privacy, which is the information leakage rate between input and output. MI considers the statistical behaviour of the consumers’ total load. Hence, it can be considered to be a more accurate privacy measurement metric. The information leakage rate calculates the reduction in the UP’s uncertainty or entropy of the consumers’ energy consumption. The value of MI naturally represents the closeness of two variables. Most approaches that protects against the attacks from strong adversaries use MI as a measure for the ability to preserve the privacy of SMs. MI is denoted by *I* and defined as follows [26,27]:

(1) $$\begin{array}{c} I\left(X^{N};Y^{N}\right):=\sum_{x_{i}\in X}\sum_{y_{i}\in Y}p\left(x_{1},y_{1},\ldots ,x_{N},y_{N}\right)\times \\ \;\log\left\{\frac{p(x_{1},y_{2},\ldots ,x_{N},y_{N})}{p\left(x_{1},\ldots ,x_{N}\right)p\left(y_{1},\ldots ,y_{N}\right)}\right\}, \end{array}$$

where $X^{N}=\left(x_{1},x_{2},\ldots ,x_{N}\right)$, $Y^{N}=\left(y_{1},y_{2},\ldots ,y_{N}\right)$. The log{·} denotes the base-2 logarithmic operation. X and Y are the possible finite set of discrete values $x_{i}$ and $y_{i}$ can take, respectively. p(·) indicates probability mass function.

*Variance:* The methods in [28,29] consider that if the SM output is constant then the signature of all individual appliances can be removed from the aggregated consumption data. The UP/adversary could not learn any information about the appliances used inside the house or any private information regarding the consumers. In [29], privacy is initially measured by load variance (*v*) defined as:

(2) $$v\overset{\Delta }{=}\frac{1}{T}\sum_{i=1}^{N}\tau_{i}{\left(y_{i}-\bar{E}\right)}^{2},$$

where *T* is the total time, and the average demand of the house $\bar{E}$ can be obtained from the history of the statistic of energy consumption in the house. The output demand $y_{i}$ (i.e., the energy received from the SG) at *i*th TS can be obtained from the SM. The time interval at *i*th TS is denoted by $\tau_{i}$, where i=1,2,3,…,N. For perfect privacy, *v* is zero. In this case, the value of the output demand $y_{i}$ for every $\tau_{i}$ must be equal to the average demand $\bar{E}$ of the house.

*Relative Entropy:* Relative entropy is also known as the Kullback–Leibler distance. It is a popular information-theoretic quantity that can be used for the comparison of two sources of information [30]. Although the relative entropy is not a distance as defined in the mathematical sense, it can quantify the relationship between two datasets. For identical datasets, the relative entropy is zero and always positive. The higher level of privacy protection, the larger the relative entropy. Relative entropy has been used in [31,32,33] to measure SM privacy. It is the distance between two probability mass functions p(x) and q(x) and is defined as:

(3) $$D\left(p||q\right)=\sum_{x\in X}p\left(x\right)\log\frac{p(x)}{q(x)}.$$

*Statistical Distance (SD)*: SD calculates the distance between two probability distributions. The smaller the value of statistical distance, the better privacy protection. SD was discussed in [12] and used in [34] for the privacy measurement of SMs. The SD between two distributions *m* and *n* for a set of *Q* can be written as:

(4) $$▵\left(m,n\right)=\max_{D\subseteq Q}(|Pr\left[m\in D\right]-Pr\left[n\in D\right]|).$$

*Matthews correlation coefficient:* To effectively prevent the occupancy detection, the Matthews correlation coefficient [35] is a standard measure of a binary classifier’s overall performance of a threshold-based attack on the home’s SM data. The lower the value of the Matthews correlation coefficient, the better the privacy protection of SMs. This metric is used in a combined heat and privacy approach [36,37].

*Fisher information:* Fisher information [38] measures the information about an unknown parameter obtainable from a sample. It informs the ability to measure a parameter, given a certain amount of data. More specifically, Fisher information quantifies the expected amount of information given by a random variable for a parameter of interest. The concept is related to the law of entropy as both aim to quantify the disorder in a system. Fisher information as a measure for privacy in SM was used in [39].

*Inferential Privacy:* Inferential privacy [40] measures the uncertainty intrinsic to human behaviour and device models. It is the probability of error when an adversary attempts to infer private information. It assumes a strong adversary model and provides a guarantee on when the adversary can infer private information about the consumers. The definition of this metric is closer to the concept of equivocation metrics [41].

Among all the aforementioned privacy metrics, MI is the most popular metric used in the literature. MI between two random variables measures the information shared by them. It measures the extent of which by knowing one variable reduces the uncertainty of the other variable. If two variables are independent, then knowing one variable does not give any information about the other variables; hence, the MI between them is zero. However, if two random variables are identical, then the information conveyed by one variable is shared with the other variable. In this case, one variable fully determines the other variable. Consequently, the MI between identical random variables is the uncertainty contained in any one of the variables. The MI between two variables quantitatively measures the amount of common information embedded within two variables regardless of the algorithm (linear or non-linear, deterministic, or statistic) used to generate one variable given the other.

#### 2.6.2. Cost Analysis Metrics

The costs for preserving SM privacy are critical in determining the practicality of implementing a privacy-preserving model. Methods that are too expensive to implement are unsuitable for consumers. In this section, we discuss some of the popular cost analysis metrics used in the literature as categorized by (i) communication costs, (ii) computational costs, and (iii) monetary/financial costs.

*Communication costs:* Communication costs can be denoted as communication overhead (CoO). CoO is mainly related to wireless reception and transmission. It includes receiving and transmitting the information bit, the analogue and digital circuits to process them, and all the radio parts involved. It also refers to the total number of exchanged messages during the execution of the protocol. All privacy preservation models that use wireless communication for data transfer incur CoO. In powerline communication (PLC) [42], privacy-preserving models use existing communication infrastructure, lowering the deployment costs. However, they have some limitations, such as interference, complex routing, and signal loss.

*Computational costs:* Computational costs are also known as computation overhead (CO). CO is related to the execution of cryptographic algorithms on the embedded platform. It also includes the bitwise/arithmetic functions, defined functions, pseudo-random number generation function, hash function, and symmetric encryption. Wireless approaches consume energy from the battery for communication and computation. The unit of measurement is the energy required to receive and/or transmit one bit of information, typically denoted in nJ/bit.

*Monetary/financial costs:* Energy bills can be reduced by strategically using the energy from the energy storage devices as shown in [29,43]. For example, when using an RB for demand-side energy management to reduce the energy bill, the RB can charges more during the lower unit price of energy (off-peak period) and discharge more during the higher unit price of energy (peak period).

When RES is used for demand-side energy management, the consumer can use the energy generated from the RES during the peak period and sell the energy back to the SG when more renewable energy is generated. When thermal energy storage and real/reactive power are used, the consumer can manage the demand by using less energy from the SG during periods with higher unit price to reduce the cost of energy. Cost savings (CoS) can be defined as follows:

(5) $$\mathrm{CoS}=\sum_{i=1}^{n}x_{i}\cdot p_{i}-\sum_{i=1}^{n}y_{i}\cdot p_{i}$$

where $x_{i}$ is the real energy demand of the house at *i*th TS for i=1,2,3,…,N, $y_{i}$ is the SM reading at *i*th TS, and $p_{i}$ is the unit price of energy at *i*th TS.

Both CoO and CO are important for when considering the practical implementation of privacy-preservation methods. Typically, the lower the CoO and CO of a particular method, the higher the possibility of the practical implementation of the approach. When RB or RES are considered, most methods considered monetary/financial cost reduction as their primary cost analysis metric.

## 3. Privacy-Preservation Techniques against Weak Adversaries

In this section, we discuss SM privacy-preservation techniques against weak adversaries as published in the literature. We classify them into two categories: (i) with and (ii) without the presence Trusted Third Parties (TTPs). In the first category, we consider privacy protection against (i) SM data alteration and (ii) Eavesdropping in communication. In the second category, in addition to the two threats without TTP, we also consider threats in (i) residential RES prediction, (ii) unreliable operation, and (iii) the energy consumer network.

### 3.1. With Trusted Third Parties

#### 3.1.1. Smart Meter Data Alteration

SM privacy can be successfully preserved by altering its data. The alteration of SM data can be performed by using techniques involving cryptography, data aggregation, and the addition of random noise. Individual SM data are observed by the UP for billing, DR program, and energy theft detection. Below, we describe the different methods that use TTP for SM data alteration.

*SM privacy for billing:* A SM reading aggregation and billing protocol that uses Paillier’s homomorphic encryption and verifiable secret sharing was proposed [44]. This method can achieve both encrypted data randomizing and data aggregation. The forward secrecy of a participant’s session key is obtained with the randomized session key generation. However, the SMs are not authenticated for data aggregation. A fake/dishonest SM may falsify the data, leading to incorrect results of the aggregation process.

The scheme does not ensure data integrity and consumer privacy. A protocol that combines in-network data aggregation and SM billing was proposed in [45]. Data aggregation enables energy supplier to balance and allocate resources, and SM billing enables dynamic pricing scheme based on fine-grained consumption profiles. A homomorphic commitment is used with the HE scheme. In the communication network, the data sent from a SM is either an encrypted message or a commitment. The method assumes that there is only a single recipient of the aggregated consumption data without considering other entities in an open electricity market (e.g., grid operators and suppliers). These entities are also authorized to access the aggregated consumption data of different sets of users.

*SM privacy for DR:* In [46], a privacy-preserving and efficient DR program that uses HE was presented. It uses an adaptive key evolution to ensure a secure forwarding of consumer session keys. However, public-key schemes, such as HE, increase overheads, which makes them unsuitable for peer-to-peer and online social networks. These schemes can secure gateways under the honest-but-curious model. In this model, the gateways honestly follow the communication protocols agreed upon among the ones involved. However, they may snoop on users’ electricity consumption out of curiosity. In reality, a gateway may become malicious due to various reasons. When there is a large number of SMs in the community, the program does not perform as efficiently when compared to the technique presented in [44].

*Differential privacy for DR:* A fault-tolerant SM protocol was proposed in [47]. The method overcomes the general communication failures and ensuring DP with lower errors and significantly improved efficiency. The protocol prevents fail-stop faults by designing cipher-text and distributing trust among the SMs (secret key sharing). However, the effect of a fault-tolerance scheme based on future cipher-texts is not ideal due to its limited buffering capacity. In this approach, the individual consumption report is aggregated in the gateways or collected before delivering to the operation centres. Hence, it cannot support dynamic billing and prevent impersonation attacks. The scheme also has practical issues, for instance, SMs may not be able to communicate with the aggregator when there is a communication failure. The method assumes that there is a secure channel between the SMs and third-party aggregator, which is not practical in the real-world.

#### 3.1.2. Eavesdropping in Communication Networks

Attackers can obtain information about the consumer by eavesdropping on the communication channel. The SG communication framework consists of the communication network between energy consumers and the UP. Different communication protocols have been proposed to preserve the privacy of SMs between energy consumers and the UPs, which is discussed as follows.

An identity-based encryption and signature scheme was presented in [48]. In this method, an SM can anonymously access services provided by the service provider using one private key without the help of the trusted anchor. This scheme also supports SM anonymity and mutual authentication. However, the authentication scheme fails to provide strong credential’s privacy of SMs. It is insecure when ephemeral secret leakage attack occurs. The scheme fails to provide the session key security and SM credential’s privacy under the widely-accepted Canetti–Krawczyk adversary model [49,50]. The method requires high communication and computational costs, which make it less suitable for the resource-constrained SMs.

A privacy-enhanced data aggregation scheme that prevents internal attacks was presented in [23]. The scheme uses blinding factors to create blinded data to prevent internal attackers, such as UPs from learning the electricity consumption of users. The UP only receives the total electricity consumption of the neighbourhood without knowing the consumption of individual users. However, by observing the user’s registration procedure, each user’s private key can be easily derived from the information published by the aggregator.

A lightweight lattice-based homomorphic privacy-preserving aggregation scheme was proposed in [51]. This scheme enables smart appliances to aggregate their readings without the help of SMs. The SM or the intermediate base station can validate the messages authenticity, although they cannot decrypt this aggregated consumption. The scheme investigates the impact of different smart appliances on HAN overhead. The total computation and communication load is distributed to different parties, such as SMs, smart appliances, and the base station. The model adopts a lightweight lattice-based homomorphic cryptosystem, which depends on simple multiplication and addition operations.

This scheme guarantees consumer privacy, data integrity, and message confidentiality. It also ensures lightweight computation and communication overhead. However, these HE-based methods incur high computational costs.

Another lightweight lattice-based secure and privacy-preserving approach for consumer clusters within a residential area was proposed in [52,53]. The cluster connection with the UP is limited only when the cluster needs to adjust its total demand. The model utilizes the Number Theory Research Unit (NTRU) cryptosystem to reduce the computational complexity [54]. However, these methods need to fully trust an entity known as a trusted authority. A privacy-preserving, efficient, decentralized, and selective aggregation approach was proposed in [55].

This approach uses a multi-recipient system model, Homomorphic Paillier encryption, and selective aggregation to prevent the SM data from internal and external attacks. It aggregates data at the gateway that is nearest to the data aggregator to conserve bandwidth and reduce the risk of creating a performance bottleneck in the system. It uses a batch signature and short signature verification to reduce computation and communication overhead. However, the customer distribution of each supplier could not be achieved. Its data collection schemes are designed to collect the total consumption of the AMI network’s consumers and cannot handle either the multidimensional nature of power consumption or multi-subset data aggregation.

A privacy-preserving metering protocol was presented in [56]. This protocol uses a distributed authentication model to speed up the message authentication process. The data collection unit does not store decryption keys for the encrypted SM data with this protocol. Hence, the original signature is transmitted to the Advanced Metering Infrastructure (AMI) server. The adversary cannot decrypt the encrypted meter data and cannot modify the SM data. Due to the decentralized message authentication mechanism, the authentication time is shorter. The protocol does not include security features, such as perfect secrecy, strong SM credential privacy, offline password guessing attack protection, password and biometric update phases, and dynamic SM addition. It incurs high communication and computational overheads, thus, resulting in performance degradation. The scheme does not support consumer anonymity and data aggregation.

A trusted SM approach was presented in [57]. It uses attribute certificates to hide the platform configuration information of an SM. A cryptography-based ring signature is used to hide user sensitive information. Using the Rivest, Shamir, and Adleman (RSA) ring signature [58], the trusted SM provides a list of properties, such as anonymity, correctness, and unforgeability. SM attributes are protected by using the digital signature.

The method can detect whether a software update is trusted or not. It can provide security against private information leakage with efficiency. A ring signature scheme is used to preserve the SM privacy at the data concentrator. However, a faulty or a compromised SM cannot be detected since the identity of a (compromised) SM is hidden in the ring. Whenever a trusted SM needs to assert its real identity to the data concentrator, it must include neighbouring trusted SMs using their public key certificates and then compute a ring signature.

Table 2 presents a summary of the SM privacy-preservation techniques discussed in this section, where the SM is faced with weak adversaries that attempt to compromise its privacy.

### 3.2. Without Trusted Third Parties

Consumers need to be able to fully trust a third party when deploying TTP-based privacy-preservation methods. However, these parties may not always be trusted. To overcome this challenge, SM privacy-preservation methods without relying on TTP have been proposed. In the following sections, we discuss how these methods could be employed to preserve the privacy of SMs, and protect against potential privacy threats.

#### 3.2.1. Predicting Household Renewable Energy Sources

The UP can predict the household demand by observing the energy generation by the RES and the SM reading/consumption of the household. The technique presented in [20] uses *Differential Privacy* for RES-based smart metering system. This method calculates the total energy consumption of the house obtained from the SG, solar energy, and wind energy. The Laplace noise is selected (when the energy consumption of houses is not zero) based on the maximum allowed error with the output, standard deviation, and variance.

The SM reading is equal to the Maximum Reported Peak Load (MRPL) if the sum of noise and consumption is more than the MRPL. Otherwise, the SM reading is the sum of the noise and the demand. In this model, information is gathered from different resources and aggregated to calculate the magnitude of the Laplace noise. It also caps the value to a specific peak value and transmits it to the UP via a wireless medium. However, the billing process of the method is very complex, and it incurs high cost overheads.

**Table 2:** Summary of SM privacy-preservation techniques against weak adversaries with TTP.

Ref.	Goals	Limitations	Metric	Mechanism
[48]	Authentication and anonymous key distribution scheme for SG communication	Fails to provide strong credential privacy, Insecure for the ephemeral secret leakage attack	CoO	ID-based encryption signature
[23]	Privacy-enhanced data aggregation against internal attackers in SG communication	Consumer private key can be derived from the published information	CO	Secure AgG
[44]	Privacy-preserving data aggregation, efficient and secure billing of SMs	SM is not authenticated during data aggregation	CO	Paillier’s HE
[45]	Privacy-preserving data aggregation and verified billing of SMs	Consider only a single recipient for aggregated data	CO	HE
[46]	Privacy in the demand response (DR) program	Unsuitable for P2P and online social network	CCO	HE
[59]	Privacy-preserving incentive-based DR program	The bidding process is not supported	CO	ID-committable signature, Partially blind signature
[47]	Differential privacy (DP) of SMs for the DR program	No dynamic billing, vulnerable to an impersonation attack	RMSE, Mean relative error	Secure AgG
[60]	Privacy of SMs for energy theft detection (ETD)	No dynamic billing, assume an online trusted entity	Cross entropy, Accuracy score	HE
[51,61]	Consumer privacy, data integrity, and message confidentiality in SG communication network	No discussion about the monitoring and detection of any intrusion activity	CCO	Lightweight lattice-based HE
[52]	Lightweight lattice-based secure and privacy-preserving communication scheme for a cluster of consumers	Employees within the system could easily access records to compromise the system	CCO	NTRU cryptosystem
[55]	Privacy-preserving and preventing users’ usage data from internal and external attacks in SG communication	Cannot handle either the multidimensional nature of power consumption or multi-subset data aggregation	CCO	Paillier’s HE, Batch signature, Short signature verification
[56]	Lightweight and privacy-preserving SM communication protocol by a distributed authentication method	Cannot protect DoS attack, incur high computational and communication costs, cannot ensure anonymity	Communication delay, Authentication time	Paillier’s HE
[57]	To hide the platform configuration information of SMs and user sensitive information for communication	Data concentrator cannot detect the compromised SMs	CoO, Time complexity	Cryptography-based ring signature

#### 3.2.2. Smart Meter Data Alteration

Different methods, such as those based on cryptography, data aggregation, and the addition random noise, have been used to alter SM data to preserve its privacy. Below, we consider several methods for SM data alteration without relying on TTP.

*SM privacy for DR:* A threshold-based anonymous identification scheme for overload audit and privacy preservation in SG was proposed in [62]. Consumer privacy depends on the power consumption in the presence of DR request from the UP, which defines an acceptable consumption threshold when there is a power shortage. The consumption model is built so that a threshold value can be found to fill the gap between power consumption and generation during a power shortage. Consumers’ power consumption should be lower than the threshold value. To meet the threshold, consumers must follow the instructions to control their consumption. Consumers who follow the instructions from the UP will maintain their anonymity whereas those who do not follow cannot preserve their privacy.

A distributed and multi-unit privacy guaranteeing bidding mechanism for DR program was proposed in [63]. This model protects the bidding information of participant, whereas the only the winner is exposed to the UP. In this scheme, customers information is disclosed neither to the bidder nor the UP. The model provides a security analysis of privacy-assured bidding by using four Zero Knowledge Proofs (ZKPs). In this protocol, the computational hardness of discrete algorithm proble, and efficient ZKPs replaces the TTP.

*Random noise-based SM privacy:* The addition of random noise can preserve the privacy of SMs. Random noise is added either from within the SM or by a TTP [64]. A lightweight, efficient, and distortion-based privacy-preserving metering and billing scheme was proposed in [65]. Random noise is added to distort the SM data so that data recovery becomes difficult for the adversary. Using prior knowledge about the added random noise and power consumption data, the scheme uses an algorithm for power consumption distribution reconstruction. It is required for prediction, dynamic pricing, and power demand analysis. However, the scheme suffers from difficulties in reconstructing original data and billing accuracy. Another technique, as presented in [66], preserves SM privacy by using both noise and cryptography-based techniques. Customers need to send the actual usage data to the UP; hence, this method is only effective against a weak adversary.

*Differential privacy of SMs:* In [67], the SM reading of consumers is arranged as a matrix for DP of SMs. The row is used for billing and the column is used for the utility. The method uses Laplace distributed random noise to preserve the privacy of SMs. The variance of the distribution is defined in such a way so that the aggregated value of the SM reading is within the maximum allowed error. This approach has a trade-off between the privacy and the number of SMs for which different statistics can be calculated at each time instant. However, a large group of SMs is needed to maintain privacy and statistical accuracy simultaneously.

Direct perturbation of power measurements may reduce the performance of power system controls and result in inaccurate billing. In [68], DP for real SM data was proposed. However, this method requires a large number of SMs for it to be effective. The SM privacy preserving method presented in [69] supports DP, data aggregation, range-based filtering, and fault tolerance simultaneously. This model is an extension of the lifted ElGamal encryption [70] that aggregates consumer consumption reports at the gateway.

It reduces the communication overhead and supports fault tolerance of malfunctioning SMs effectively. To filter abnormal measurements caused by false data injection attack or electricity theft without exposing consumers measurements, the model uses zero-knowledge range proof. Since individual consumption reports are aggregated at the gateways, the scheme could not not support dynamic billing. The ElGamal encryption-based schemes are also computationally expensive.

*Privacy in energy theft detection:* A privacy-preserving energy theft detection scheme was proposed in [71]. To detect abnormal data and estimate the customer energy consumption, a recursive filter based on state estimation is used. For data transmission, the model uses a lightweight NTRU algorithm [54] and signature authentication to protect the privacy of SM data. However, the approach tampers with SM data. Hence, accurate state estimation may not be possible, and the billing process can also become quite complex.

*Information-theory-based privacy of SM:* The use of information-theoretic measures is a commonly used approach to model privacy loss by using optimally-designed noise under certain utility constraints. An information-theoretic framework was presented in [72]. It considers a privacy-utility trade-off problem with the minimum assumption that it is traceable. Tools from information theory and a hidden Markov model are used.

The theory of rate-distortion is used to quantify the trade-off between privacy (information leakage) and utility (mean square distortion). For an electric load of a stationary Gaussian model, the optimal solution exploits the presence of high-power but less private appliances. It then filters out frequency components with lower power relative to a distortion threshold. However, it is challenging for the model to handle physical constraints. This approach enforces privacy in a probabilistic manner, which means there may be a severe breach of privacy for a rare realization of the system. In [72], the impact of techniques, such as noise addition and down-sampling, are not analysed in terms of privacy breach and data utility. Finding a solution to address this issue may help consumers to choose between load scheduling-based solutions and low-cost data manipulation techniques.

For privacy preservation, the mechanism in [73] allows energy customers to flexibly down-sample their energy usage data before sharing it with third-party service providers. This is performed without invalidating data issuer’s digital signature. This method lacks a quantitative evaluation of the trade-off between data utility and privacy. This method could also limit the utility of the SMs data for certain applications requiring a timely response.

*Statistical privacy of SM:* Two schemes that collect aggregated statistics while preserving the privacy of electric consumers were presented in [74]. The first scheme supports dynamic profiling that can extract statistical information without compromising consumer privacy. It enables the aggregator to efficiently extract the summation of the information from the received consumers’ responses. The second scheme can extract correlation information among various factors for the smart system design. It can also be used as an underlying tool for association rule mining and baseline inference. Although the aggregate statistics are effective in concealing detailed usage profiles, the correlations involved are not perfect. It is not as effective as statistical models derived from actual individual household energy consumption data.

*Blockchain-based SM privacy:* To preserve the privacy of consumers in SGs, a data aggregation scheme using Blockchain was proposed in [75]. This scheme divides the consumers into different groups. Each group has a private Blockchain that records the members’ data. To hide the users’ identity, it uses pseudonyms that preserve inner privacy of the user. The system model consists of Neighbourhood Area Networks (NANs) and Wide Area Networks (WANs). For aggregation, SMs send the data to the mining node, and the aggregated data of each group is sent to the WAN. The mining node records the aggregated data into a private Blockchain and publishes them for verification. For fast authentication, a bloom filter is adopted in the system. However, the computational complexity of this method is very high.

A technique using consortium Blockchain was proposed in [76]. In this method, an elliptic curve point multiplication operation is used in the certificate-less aggregated ring signcryption algorithm. It reduces the computing and communication costs. Distributed storage of users’ data with a consortium Blockchain is used to solve the problem of single-point failure and data tampering. However, the use of excessive energy is required for practical implementation. The transaction validation time and storage requirements are a challenge in blockchain-based methods.

Table 3 presents a summary of the SM privacy-preservation techniques for RES-based households by altering the SM data to defend against weak adversaries.

#### 3.2.3. Eavesdropping in Communication Networks

In this section, we consider privacy-preservation techniques that protects against eavesdropping in advanced SG networks, where communication links are established between energy consumers and the UP.

*Communication between energy consumers and the UP:* A privacy-preserving recording and gateway-assisted authentication scheme was proposed in [77]. In this model, the UP does not know which user requests for more power or is in agreement of using less power until the consumer uses it. After the requested power has been consumed (or by the end of each billing period), the user can prove to the UP that the energy has indeed been requested. This method uses gateway SMs to aggregate power usage information. The power generator determines the required total power to be generated at different times. This helps to reduce the total traffic volume in the communication network.

Gateway SMs also help to filter messages before reaching to the CC, hence, reducing the impact of attacking traffic. However, the use of HE in the method is a compute-intensive operation. The gateway acts as a trusted role to provide the aggregated data to the private key holder, such as a CC. The gateway provides single cipher-text to the individual consumer. Since the single cipher-text is decrypted, individual privacy can be deduced. The authentication of an SM is performed at the NAN gateway. However, it cannot validate whether the NAN gateway is a legal entity. In addition, a house blackout can be performed by a fake control command.

**Table 3:** Summary of SM privacy-preservation techniques for RES-based SM and data alteration against weak adversaries without TTP.

Ref.	Goals	Limitations	Metric	Mechanism
[20]	DP for RES-based smart metering system for the privacy of RES-based house-hold	Complex billing, inaccurate aggregation output, trace of renewable energy added by UP	Graphical comparison	Laplace Noise
[62]	A privacy-preserving threshold-based anonymous identification scheme for overload audit in DR	Consumers need to follow the instruction to curtail their consumption	CCO	Threshold value
[75]	Consumer privacy by using blockchain	Computational complexity is very high	CCO	AgG, Pseudonyms
[63]	Distributed and multi-unit privacy-preserving bidding mechanism for DR	Computational and overhead complexity is very high	NA	ZKP
[64]	Privacy model for an SM application by using random noise	Need to trust either UP or TTP	Confidence intervals	Random bit, AgG, Enc
[65]	Lightweight, efficient distortion-based privacy-preserving metering and billing	Suffers from billing accuracy and difficulties in reconstructing original data	Graphical comparison, NILM	Gaussian noise, AgG
[66]	Random noise-based privacy preservation of consumers, sharing energy usage data with third parties	No use of real SM data for performance analysis	NA	Random noise, Cryptography, ZKP
[67]	DP for consumer’s billing and utility	A large group of SMs are needed to maintain privacy and accuracy simultaneously	Graphical comparison, CO	Laplace Noise
[68]	DP for real SM data	A large number of SMs are required for useful data	Relative error	Laplace Noise
[69]	SM privacy that supports data aggregation, range-based filtering, DP, and fault tolerance	Costly computation, cannot support dynamic billing	CCO	EIGamal encryption, ZKP
[71]	Privacy for ETD, state estimation, and abnormal data detection	Tamper the SM data, the complex billing process	CCO	NTRU, Signature authentication
[72]	Information-theory-based privacy-utility trade-off problem with minimum traceable assumption	Difficult to handle physical constraints, enforces privacy in a probabilistic manner, which means there may be a privacy breach	MI	Theory of rate distortion
[73]	Privacy-preserving energy usage data sharing by using information theory	Lacks of quantitative evaluation of data utility-privacy trade-off	CCO	Flexible down-sampling
[74]	Privacy of SMs by aggregated statistics	Correlations involved are not perfect	CCO	AgG
[76]	Privacy by using consortium blockchain	Excessive energy is required for the practical implementation	CCO	Certificate-less aggregated ring signcryption

A quantitative mean was presented in [78] to identify a trade-off between the precision on the aggregated measurements, the aggregation set size, and the privacy level. This model first defines an attack on the privacy of each consumer. Coloured noise and individual SM data are aggregated to the success probability of the attacks. The approach uses data perturbation and provides formulas to calculate the ϵ privacy in the case of coloured and Gaussian white noise. By suitably colouring the noise, the model gains the same level of privacy with a much lower noise variance. However, tampering with SM readings before transmission to the UP reduces their relevance for billing purposes.

Unreasonable random obfuscation values chosen by consumers reduces the utility of the data and impacts the accuracy of the aggregated result. To set an obfuscation value for each user, it is necessary to design a reasonable random number generation algorithm. In [79], a scheme that constructs a linkable anonymous credential protocol using a Camenisch–Lysyanskaya signature [80] was presented. The method uses the message security properties, such as authentication and traceability of fault smart metering, to provide privacy protection.

Due to some other properties, such as no need for TTP, dynamic user enrolment, and revocation, the computational cost and communication overhead of the scheme is lower. However, it takes advantage of the storage and computation capabilities of cloud computing as well as the distributed and low-latency properties of fog computing. The method requires a large computation capacity, which is not always available in real-world SGs.

A privacy-preserving approach for the spatio-temporal aggregation of time series data was presented in [81]. The approach uses a masking scheme to obfuscate individual consumption and generate the correct results upon aggregation. The model can be used for any time-series data and focuses on a high degree of error-resilience that is crucial for the distributed nature of the SG.

To preserve consumer privacy, a meter data obfuscation approach was proposed in [82]. This model proposes two secure obfuscation value distribution mechanisms using IEEE 802.11-based wireless mesh networks. By using obfuscation values, the meter reading is obfuscated to protect consumer privacy while enabling UP to use the data for state estimation. For performance evaluation, the model uses a large scale AMI network that builds upon the IEEE 802.11 wireless mesh standard. However, the method requires a higher setup and computational cost and, thus, is inefficient in terms of transmission and computations.

An energy consumption prediction framework, called “Seer Grid” was proposed in [83]. The framework aims to reduce the trade-offs between data utility and privacy. The first-level prediction is performed by each SM at the household level. Instead of using actual energy consumption data, the predicted energy consumption pattern is reported to the Cluster Head (CH). The second-level prediction is at the neighbourhood level and performed by the CH. Energy spikes are predicted by the CH in the cluster or neighbourhood and shared with the UP.

The model is designed so that it maintains the correlation between the actual energy and the predicted consumption patterns at the cluster level. It removes the correlation in the predicted data communicated by each SM to the CH. The method maintains the utility of the cluster-level energy consumption data communicated to the UP. It preserves the privacy of the household-level energy consumption data against the UP and the CH. However, the framework does not formulate and generalize the minimization of privacy-utility trade-offs.

A privacy-preserving scheme for long term evolution or hybrid AMI was presented in [84]. In this scheme, the UP gathers the power bids from the consumers in a privacy-preserving and secure manner. To preserve privacy, the model uses HE so that the UP cannot correlate the consumer bids. First, the UP wants to buy power from the users. The UP generates a packet that contains a signature and purchase price. The UP sends it to the users by using the aggregator node that utilizes the backhaul network.

Next, the aggregator checks the integrity and authenticity of the packet after receiving them. If the result is true, the aggregator sends the packets to the users. The SM verifies the integrity and authenticity after receiving the packet and sends a reply-packet that contains the amount of power unit, price, and a signature back to the aggregator node.

The aggregator then gathers the reply-packets from all users and aggregates them in a secure way (using HE and a signature) and send the bid-packets to the UP. Finally, the UP verifies the integrity and authenticity of the bid-packets. The individuals’ bid cannot be correlated at the UP. However, this approach does not consider real-time monitoring systems. It mainly focuses on authentication without considering the confidentiality of SM data. It incurs high communication costs and lacks threat modelling.

Privacy and security concerns for distributed demand management protocols were highlighted in [85]. The model presents a mutually exclusive cluster-based solution to ensure privacy, confidentiality, and integrity among the users’ sharing information. The model also includes a distributed multi-party computation protocol based on clustering. The protocol can identify untruthful users in the network. A verifier-based solution is used to detect malicious nodes who declared false information about its usage. In this model, data aggregation ensures that only the overall power usage data is known by others.

However, the UP requires the calculation of some specific information, such as load forecasting and dynamic pricing. In the case where two neighbouring SMs collide in the ring, SM data could be easily disclosed. The approach in [86] quantitatively measures the information leakage of certain appliances status from SM readings and defines a privacy notion to bound the information leakage. The scheme involves a privacy-preserving algorithm to stream output readings without any data aggregation and assures utility and privacy. The outputs can support different SM services, such as regional statistics, billing, and load monitoring.

These outputs can also be fed into aggregation-based solutions. The techniques can be tailored to allow micro-grids pre-process their metering data (i.e., consumption and generation), and the main grid to solve optimization problems in real-time. However, significant utility loss can occur when metering data obfuscation, and the computational load significantly increases [87,88,89].

A privacy-preserving secure SM data-collecting protocol for calculating transmission/distribution data, and imbalance fees was proposed in [90]. The model uses secure multi-party computation as the underlying cryptographic primitive to preserve privacy. Three different data aggregation algorithms are used by the scheme that offers different balances between privacy-protection and performance. The model uses a threshold-based secret sharing scheme to mitigate collusion attacks. A realistic scenario is considered where the SM data requires to be sent to different parties, such as suppliers and grid operators.

The scheme claims to accurately calculate the distribution, transmission, and grid balancing fees. However, it does not cover the entire asset and services sharing process. It does not fulfil any data protection regulations (although it is privacy-preserving from a technological perspective) and does not consider incentive-based economic analysis.

Table 4 presents a summary of the SM privacy-preservation techniques for defending against eavesdropping in the communication networks between household consumers and the UP.

#### 3.2.4. Unreliable Smart Grid Operations

Unreliable operations in the CC can breach the privacy of individual consumers; hence, access control in SG operations is crucial. Below, we summarize the state-of-the-art solutions for access control without relying on TTPs.

*Access control:* A privacy-preserving data aggregation and access control in SG was proposed in [91]. The aggregation of SM data is performed in HAN, BAN, and NAN so that the privacy of the consumer is preserved using HE. The collected consumer data is sent to the substation and observed by the Remote Terminal Unit (RTU). The use of attribute-based encryption allows selective access to consumer data, which is stored in data repositories and can be used by different SG users. The RTU, users’ cryptographic keys, and attributes are distributed by different key distributor centres. The RTU sends encrypted data under a set of attributes.

The RTU is managed in the substations and protected in the control room, and so it is assumed to be trusted. Consumers who have valid attributes can decrypt the information provided. The access control scheme is distributed. It does not rely on a single key distributor centre to distribute the keys, which makes the approach robust. However, the method is limited by their required computational or communication complexity. The scheme cannot meet the demand when the control centre needs to determine the distribution of electricity consumption. The adversary can decrypt the encrypted measurements if it has access to the private keys of the suppliers.

**Table 4:** Summary of SM privacy-preservation techniques for communication protocols against weak adversaries without TTP.

Ref.	Goals	Limitations	Metric	Mechanism
[77]	Privacy-preserving recording and gateway-assisted authentication scheme	The scheme may lead to many security threats, assume a trusted gateway	CCO	HE, Bloom filter
[78]	A trade-off between the privacy level, precision on aggregated measurements, and the aggregation set size	Random obfuscation value may impact the accuracy of the aggregated result	ϵ-privacy	AgG, Gaussian white and coloured noise
[79]	SM privacy-preserving scheme using the linkable anonymous credential	Use the advantages of the storage and computation capabilities of cloud computing and the distributed, low-latency properties of fog computing	CCO	Camenisch–Lysyanskaya signature
[81]	Privacy-preserving scheme for Spatio-temporal aggregation of time series data	Complicated to implement and computationally expensive to execute	CCO	AgG, Masking
[82]	Consumer privacy and distribution state estimation using data obfuscation	Requires higher setup and computation cost	Goodput, Data delay, Packet delivery ratio	Secure obfuscation value distribution
[83]	Reduce the trade-off between data utility for the UP and privacy for the consumers	Does not formulate or generalize the minimization of privacy-utility trade-off in Seer Grid	CCO	Two level prediction
[84]	Privacy-aware scheme over hybrid advanced metering infrastructure	Does not involve a real-time monitoring system, focus on authentication without considering the confidentiality	CCO, Data delay, Packet delivery ratio	HE, AgG
[85]	Privacy and security issues for the distributed demand management protocols	SM data could be learnt if two neighbouring SMs in the ring collude, no consideration of load forecasting	Execution time, Cost of energy	Game theory, Multi-party computing
[86]	Privacy-preserving algorithm to stream SM readings without any aggregation while ensuring utility and privacy	Significant utility loss by SM data obfuscation and greatly increase the computational load for the SG	Graphical comparison, Error rate, CO	Streaming algorithm
[90]	Secure and privacy-preserving protocol to collect operational SM data needed to calculate transmission, distribution, and imbalance fees	Not fully General Data Protection Regulation compliant, not cover the entire asset and services sharing process	CCO	Threshold-based secret sharing, Secure multi-party computing, AgG

A game-theoretic approach was proposed in [92] to strike a balance between consumer privacy and the benefits of using SM data in a deregulated SG environment. The access control problem is solved by fairly compensating users for their participation in the data market by using the concept of DP. First, the model defines the privacy risk depending on the sensitivity level of private information. Next, a game-theoretic negotiation mechanism was proposed to investigate fairness among the consumer, data aggregator, and third parties.

The goal of each player in the game is to maximize the utility. The consumers are interested in maximizing their reward from allowing access to power consumption data. The data aggregator expects higher profit margins from third parties and to provide fewer incentives to consumers. Finally, third parties expect to pay less for data and obtain higher quality and higher cardinality. However, the method incurs a cost burden on the aggregator. It does not allow consumers to be involved in the decision-making process.

To address privacy issues in access control, a multi-resolution load curve representation was proposed in [93]. The model combines multi-resolution representation with secure aggregation. For secure signal processing, the scheme enables model access on a “need-to-know” basis. Access control for the entire system is encrypted and aggregated. Access is granted to parties for individual resolutions based on their roles and the specific needs in terms of data resolution.

The multi-resolution analysis is used for the privacy-preserving protocols and directly depends on the HE property for secure aggregation. By applying HE to a signal represented in the wavelet domain, homomorphic additivity not only preserves privacy but can also be separately exploited for each resolution. However, the method does not consider the trade-off between privacy and utility for the user. It does not consider DR and cost-reduction during the privacy preservation of SMs. Table 5 summarizes the privacy-preservation methods of SMs for access control.

To conclude, SM privacy-preservation mechanisms designed and developed to defend against the attacks from weak adversaries usually involve altering the data at the SMs. These methods have drawbacks and can lead to inaccurate state estimation, complex billing process, and misleading control signals. Another challenge for preserving privacy against a weak adversary is that the models proposed often cannot be deployed directly onto the existing SG infrastructure.

Practical implementation of these methods can be costly. A particular challenge is that the UP has control over the entire distribution system, so it can install a sensor to the distribution line to learn about the energy requested by households. It is crucial for privacy-preserving mechanisms to consider such cases where strong adversaries are present and have full control of the SM data. We will discuss the relevant techniques and methods presented in the literature that aim to defend against such adversaries.

**Table 5:** Summary of SM privacy-preservation techniques for access control in operation against weak adversaries without TTP.

Ref.	Goals	Limitations	Metric	Mechanism
[91]	Privacy protection for access control and data aggregation in SG	If an adversary gain access to the private key of the supplier and can intercept encrypted measurements, the adversary can decrypt them	CCO	AgG, HE
[92]	Game-theoretic approach for access control to balance between beneficial use of SMs and individuals privacy in the deregulated SG	Does not provides consumers with a better sensibility of involvement in the decision making of their assets, has cost burden on the aggregator	Trade-off between privacy and benefits	AgG, Game theory, DP
[93]	Privacy preservation access control in operation using multi-resolution load curve representation	Does not consider the trade-off between privacy and utility, DR, and cost-reduction during the privacy of SMs	CCO, Standard deviation	Wavelet-based multi-resolution, AgG, HE

## 4. Privacy-Preservation Techniques against Strong Adversaries

Strong adversaries refer to attackers who have full access to the SM data as reported to the UP. A strong adversary can analyse users’ SM data, and by using the historical data, the adversary can determine private and sensitive information about consumer household activities. To protect the consumers’ privacy against strong adversaries, advanced privacy protection mechanisms are needed to mask any information from the SM output. The most effective way to achieve this is by using demand-side energy management.

Demand-side energy management can be performed by using energy storage, such as a Rechargeable Battery (RB), Renewable Energy Sources (RES) (such as solar panels and wind turbines), HVAC systems, and reactive power storage. By using energy from energy storage devices, the EMU alters the actual energy requested by the households.

For example, the SM does not record the actual energy consumption of the house by using demand-side energy management. It records the energy consumption data, which has already been strategically masked. If an adversary tampers with the SM data, it will not compromise the privacy of the consumers. Similarly, information obtained from the communication network or operation centre will not compromise the privacy of the consumers.

In this section, we categorize demand-side energy management schemes into two categories: (i) prioritizing customer privacy and (ii) prioritizing cost reduction (cost-friendly) schemes.

### 4.1. Prioritizing Customer Privacy

In this section, we discuss demand-side energy management methods that prioritize customer privacy. These methods mask the actual energy consumption of the consumer by strategically using energy from energy storage devices.

#### 4.1.1. Using Rechargeable Batteries

RB can be used to mask energy consumption in the house. To mask the actual energy consumption, the RB can charge during the off-peak periods (lower demand of the house) and discharge during the peak periods (higher demand of the house). Below, we describe several well-known methods to achieve this goal.

*Heuristic methods:* The first RB-based policy to preserve the privacy of SMs was proposed in [31]. This method attempts to hold the output load to its most recent value by charging and discharging the RB. If the previous load is higher than the current load, the RB charges to make the current output load equal or near to the previous load (if there is enough room in the RB). However, if the previous output load is lower than the current load, the RB discharges (if there is enough energy in the RB to make the output load equal or close to the previous output load). This model is known as the “best effort” method. However, this method is unable to preserve privacy when there is a continuous increase or decrease of appliance demand for a longer period, or when the State of Charge (SoC) is too low or too high.

*Random noise-based methods:* In [94], it was demonstrated that Battery-based Load Hiding (BLH) methods, such as best-effort [31], non-intrusive load levelling [95], and stepping algorithms [96], do not preserve differential privacy (DP). These methods use randomized algorithms by using binomial noise (known as coarse-grained noise and fine-grained noise) to preserve DP. However, it is unable to generate the required noise due to RB constraints. In [34], a method was proposed to preserve the DP of SMs by adding Laplace distributed random noise with the input load.

The noise is generated by charging and discharging the RB. There are constraints in RBs, such as battery throughput (or maximum charge–discharge rate of an RB) and limited capacity. To generate a Laplace distributed random noise to approximate DP, this method first considers the constraints of a RB. It then considers a second RB that is connected in cascade with the primary RB for recharging.

*SM privacy for Demand Response (DR):* In [97], SM privacy issues within residential appliances and DR energy management are explored. To schedule customer appliances and RBs, an online approach based on stochastic optimization is used. Three different load-shaping strategies for masking household energy usage were investigated. The best strategy minimizes the differences between the electricity usage of each appliance at different time instants. However, the accurate estimation of the probability distributions is challenging in practice. The impact of data uncertainties on the optimal performance could not be fully captured in the stochastic optimization-based energy management schemes.

*Information theory based privacy of SMs:* An information-theory-based privacy smart metering system using cascaded RBs was proposed in [98]. In this scheme, two RBs are connected in series to reduce information leakage rate. Invariance conditions were considered for defining the upper and lower bound of the information leakage. This approach defines the upper and lower bound of the information leakage in cascaded batteries.

It considers binary and ternary input to analyse system performance. It is noted that the use of two RBs in cascade can reduce the rate of information leakage, when compared with using a single RB whose capacity is equal to the two RBs. Information-theoretic privacy of smart metering systems using an RB was studied in [99]. An RB is used to partially obscure the energy demand of the consumer. After a series of reduction of the original problem, the model shows that the most optimal battery policy to preserve privacy can be recast as a Markov decision process [100]. Consequently, the minimum information leakage rate and the optimal charging policies are provided as the solution of a suitable dynamic program.

Fisher information was used in [39] to quantify SM privacy. An optimal RB policy was designed to reduce the Fisher information by using the Cramer–Rao bound [101] so that the variance between the input and the output load is maximized. This method assumes that the RB policy does not depend on household consumption, and it considers the cost of an RB for charging and discharging. The authors demonstrated the boundary of the RB policy and the change of estimation error variance in relation to the RB capacity. However, they did not demonstrate a practical implementation of this method.

In [102], the hypothesis testing problem in SMs was analysed. In this hypothesis, consumer privacy can be preserved with the EMU. The EMU can choose two different policies. First, in the normal-mode policy, the EMU supplies the total energy from the SG for the demand of the house. Secondly, the EMU supplies some portion of energy from the grid, which is independent of the demand in the house. The RB with a limited capacity supplies the remaining demand or absorbs the excess energy.

This is to conceal the real energy consumption of the house and preserve the privacy of the consumers. However, the UP can guess the policies used; for example, the UP can distinguish the policy of the EMU by validating the SM data to be used for forecasting applications. The EMU strategy is used when the consumers have prioritized SM data privacy. This method does not address several basic issues about the SM data preservation, such as the owner of SM data, the amount of personal information that can be obtained, and the possibility of disguising data to preserve privacy.

In [103], SM privacy using RBs is considered with the co-operation of consumers. The study analysed the effect of user co-operation on enhancing the privacy of SMs. This model derives the lower and upper limit of the minimum leakage rate. They concluded that N-user co-operation reduces per-user information leakage approximately by a factor of $\frac{1}{N}$. A battery policy that is optimal for a single user is not optimal when the users co-operate. This method requires a large number of cooperative users. The study did not consider actual SM data traces for their simulation work.

#### 4.1.2. Using Renewable Energy Sources

RES (such as solar panels and wind turbines) can be used to preserve data privacy of SMs by using various information-theory-based methods. A model that defines the SM privacy preservation as a function of the average power of the RES was proposed in [104]. The privacy power function is a non-increasing convex function of the average power. For discrete load, the model shows that the output load alphabet can be restricted to the input load alphabet. For binary input load, there is a constant high and low power level.

For a continuous input load, the scheme derives a lower bound on the privacy power function with the help of a Shannon lower bound. The model also shows that optimal allocation of the energy provided by the RES is achievable when there is an independent exponentially distributed load. This model uses a reverse water filling solution for optimal power distributed to each user from the RES. However, this method does not use practical SM readings for performance analysis.

In [105], the peak and average power constraints of the RES were considered. The model achieves optimal privacy by considering the instantaneous input load with a stochastic energy management policy instead of a deterministic policy. The method uses a new conditional probability function for the new estimated output load. It proves that the output load alphabet can be restricted to be equal to the input load alphabet without the loss of optimality. By reducing the size of output load alphabet, the privacy-power function characterization becomes a convex optimization problem. However, this model only uses a binary input load, which can be challenging to deploy practically.

#### 4.1.3. Using Both RB and RES

Both RB and RES can be used for demand-side energy management to preserve the privacy of SMs. In [106], the combination of both RB and RES was considered for partially hiding the actual energy consumption of the consumers. The method considers the impact of the knowledge of the amount of energy generated by the RES at UP. It characterizes the minimum information leakage rate as a computable information-theoretic single-letter expression. This is performed by considering two extreme cases of a RB, such as when the capacity of an RB is infinite and zero.

An information-theory-based privacy preservation approach with both RB and RES was proposed in [107]. The RB can only obtain energy supply from the RES but not from the grid. This approach considers a RES with infinite RB. It is shown that achievable privacy is equal to the average power constrain scenario. The model considers both “store and hide”, and the “best effort” scheme for designing an EM policy that minimizes the information leakage rate. The study also considered using RES without RB in two scenarios.

First, it considers that the RES state is known by the EMU and obtain the information leakage rate. Secondly, they considered that the RES state is known by the UP. This is the worst-case situation, and the information leakage is more than or equal to the previous case. However, the method only used binary models for performance analysis, so it may be unrealistic for actual implementation.

A smart metering model with a RES and an energy controller were proposed in [32]. The privacy leakage of an SM was modelled as an unauthorized Neyman–Pearson hypothesis testing [108] on the private behaviour of the users. In this model, the privacy risk is measured with the asymptotic exponential decay rate of the minimum Type II error probability of the adversary. This model studied and characterized the optimal energy control strategy that satisfies energy demands and suppresses the privacy risk. However, the hypothesis was chosen at the consumer level, which does not control the EMU policy.

The privacy of SMs by using adversarial hypothesis testing was proposed in [33]. Privacy leakage is measured by using the probabilistic error in a binary hypothesis test. It attempts to detect consumer behaviour by using meter readings. The model characterizes the optimal privacy-preserving energy management with an adversarial hypothesis test in the context of SM privacy.

#### 4.1.4. Using Thermal Energy Storage

Demand-side energy management can be performed by using thermal energy storage, such as water heaters and space heaters to preserve the SM privacy. The methods presented in [37] used thermal energy storage to prevent the occupation detection of the house. The approach combined heat and privacy mechanism such that it changes the usage data in such a manner so that the house is always perceived to be occupied, hence, tricking occupancy detection techniques. However, the scheme could be problematic in the presence of demand response services.

#### 4.1.5. Using Real and Reactive Power

Demand-side energy management can be performed by varying reactive power in the reactive devices used in households. For example, capacitance can be used, which acts as storage similar to RB. The attacker can infer the appliance usage in the house by analysing the reactive power recorded in an SM, thus, posing a threat in the privacy of consumers [109]. A mechanism known as reactive power obfuscation is used to overcome this problem. The method smooths the power fluctuation by using capacitors that store and provide reactive power in a controlled manner. The ON/OFF signature of appliances are hidden from the output readings of an SM.

In this section, we discuss the SM privacy-preservation methods with demand-side energy management to mitigate threats from strong adversaries (prioritizing customer privacy more than the incurred costs). Table 6 summarizes the methods discussed in this section.

### 4.2. Prioritizing Cost-Friendliness

In the previous section, we considered various demand-side energy management schemes that do not prioritize energy costs. In this section, we discuss methods to preserve customer privacy while also providing a cost-friendly approach.

#### 4.2.1. Using Rechargeable Batteries

By strategically charging and discharging the RB, it is also possible to reduce the cost of energy consumption. Below, we describe the different privacy methods that present a cost-friendly approach.

*Heuristic methods:* An optimization equation for minimizing load variance and cost was proposed in [43,110]. The method requires the average load information, which involves knowing the future demand of a household. This method assumes that the total charge/discharge energy of an RB is zero. The battery policy for preserving privacy and reducing cost only depends on the current load and the price. This method uses Lyapunov optimization algorithm for an online control algorithm. With and without charging-discharging the RB, the approach develops two policies for cost-friendly privacy.

The scheme selects and implements the control decision, which has a minimum load variance and energy cost. However, this scheme assumes that the total expected value of battery charging and discharging must be zero, which may not always be possible in practice. There is a loss of RB capacity for each cycle of charging and discharging. Hence, the expected value of total charging and discharging may not be equal to zero.

*Random noise-based methods:* A RB-based cost-friendly DP was proposed in [111]. The method defines a modified Laplacian noise based on the RB constraints. First, the approach proposed an RB-based DP method. It then considered two cost-friendly DP methods of SMs. The algorithm is designed so that the probability of charging the RB during low unit price increases whereas it decreases during high unit price. To achieve a dynamic pricing policy, they used the regret mechanism of a multi-arm bandit for preserving DP and reducing costs. It was demonstrated that both RB-based DP and cost-friendly DP scheme preserves DP. However, the conditions they used for the probability density function to generate Laplace distributed random noise using a RB is challenging in practice.

*Information-theory-based privacy of SMs:* An RB-based privacy-preservation method using a Markov decision process is designed in [112]. The method first defines a weighted cost function to study the trade-off between privacy and cost. It then converts the privacy metric as a sum of the sequence of information leakage rate, and uses the Bellman equation for expressing the problem in a special kind of partially observable Markov decision process with belief dependent reward. However, finding a solution of Bellman equation and computing optimal stationary strategy is computationally complex especially for continuous state space. The model provides an upper bound to the optimal trade-off by using rate-distortion optimization and an inner bound by using a greedy algorithm that maximizes the instantaneous reward at each step. However, the method uses binary battery states for performance analysis, i.i.d. (independent and identically distributed) load and price model, which is challenging in practice.

**Table 6:** Summary of SM privacy-preservation techniques against strong adversaries (prioritizing customer privacy).

Ref.	Goals	Limitations	Metric	Energy Storage
[31]	To hide the load signature by a heuristic method	No privacy for continuous increase or decrease of demand	Regression analysis, cluster classification, Relative entropy	RB
[104]	Characterize optimal privacy by using information theory in multi-users	No practical use SM reading	MI	RES
[106,107]	Characterizing the minimum information leakage rate by using Information theory	Only uses a binary model for performance evaluation	MI	RB and RES
[94]	To preserve approximate DP (using binomial noise) of SMs	Less efficient due to frequent charge and discharge of an RB	MI	RB
[34]	To preserve approximate DP (using Laplace distributed random noise) of SMs	No consideration of cost reduction during DP	MI	Cascaded RBs
[97]	Optimize SM privacy for the residential appliances DR	Accurate estimation for the probability distributions of uncertain data is a huge challenge in practice	Sum of distance and Coefficient of determination	RB
[98]	Using information theory to quantify privacy leakage for cascaded RBs	Use of unrealistic load for simulation	MI	Cascaded RBs
[99]	Information-theory-based optimal RB charging policy for minimum information leakage	Use of unrealistic load for performance analysis	MI	RB
[102]	Use of HT to reduce information leakage (information-theory-based)	Did not address several basic issues about the SM data	MI	RB
[103]	Information-theory-based method where users co-operation to enhance privacy	Required a large number of users for practical implementation	MI	RB
[105]	Characterize optimal privacy by using information theory	No use of real load for simulation	MI	RES
[32]	Satisfies energy demands and suppresses the privacy risk by unauthorized Neyman–Pearson HT	The method chooses the hypothesis at the consumer level	Probability of error	RB, RES
[33]	Characterize optimal privacy by Adversarial HT	Does not consider anomaly detection	Probability of error	RB, RES
[36,37]	Combined heat and privacy mechanism to mask occupancy detection using the heuristic method	Problematic in the presence of DR service	Matthews correlation coefficient, Error factor	Thermal energy storage
[40]	Information-theory-based Optimal trade-off between the SG operations and privacy	No consideration of cost reduction for privacy	Inferential privacy	Thermal energy storage
[109]	Reactive power obfuscation of SMs by using heuristic method	No consideration of cost reduction during the privacy	Entropy, Obfuscation factor, Detection rate	Real and reactive energy storage

The method in [113] was designed with optimal energy management strategies that consider the trade-off between the privacy of a consumer and the expected cost of energy storage or a RB. The scheme shows that the problem of designing a cost-efficient and privacy-enhancing energy management strategy can be reformulated as a belief-state Markov decision process problem. The optimal solution can be derived using Bellman dynamic programming. This method uses the expected cost-saving rate and Kullback–Leibler divergence rate for performance analysis [30].

*Machine-learning-based methods:* A cost-friendly privacy method based on reinforcement learning named RL-BLH was proposed in [114]. The model hides both the high and low-frequency consumption pattern. The method shapes the SM reading to a rectangular pulse of varying magnitude. The RB charges during the low unit price of energy and discharges during the high unit price of energy to reduce energy expenditure. To learn the decision policy for controlling the RB level without any prior knowledge of consumption profile, a reinforcement learning technique was applied. By using a linear combination of a few selected features, the model approximates the optimal action-value function so that actual usage value can be taken into account without quantization. However, the convergence time of the reinforcement learning process is high.

#### 4.2.2. Using Both RB and RES

The energy generated by the RES can also be sold back to the grid during lower energy demand in the house. Hence, RES can be used for cost-friendly privacy of the SM. Cost-friendly privacy using both RB and RES are discussed below.

*Heuristic methods:* Cost-friendly privacy using a RB and local energy source is discussed in [115]. The model uses energy source with real weather prediction to preserve privacy and increase the efficiency of energy usage. The method obfuscates whether the building is occupied by someone or not. It introduces a decisional model between the RB, the RES, and the UP to optimally preserve privacy and reduce the cost of energy. In this approach, if the current demand is less than the average demand, then an output load is determined that satisfy the demand and charge a portion of the RB. If the current demand is higher than the average demand, the EMU finds a value of an output load to satisfy the demand by using both renewable energy source and the portion of the RB. The method compares the input and output curves. The method does not use any metric for performance measurements, and the policy for reducing the energy costs is not presented.

*Information-theory-based privacy of SMs:* In [116], SM privacy is studied from the fundamental theoretical perspective, and both RES and RB were considered simultaneously. A method that only uses the RES is first demonstrated. This method depends upon the average and peak power of the RES. An approach with the addition of a RB is then demonstrated. The RB be used to store the surplus energy harvested by the RES. It can also be used to preserve privacy. A finite state model is defined by using a RB and RES.

The information leakage rate is calculated by using the Trellis algorithm. It shows that with the increase of the capacity of the energy harvesting unit, the information leakage reduces while the rate of wasted energy increases. Using the Pareto optimal curve, the model obtains an optimal Pareto boundary for information leakage versus wasted energy rate. Since energy management policies are time-varying, it is impractical to consider a time-invariant policy. The maximum charge/discharge rate of an RB is not considered in designing the policy. The Trellis algorithm also does not consider the fact that an eavesdropper can use the observation to estimate historical energy consumption.

#### 4.2.3. Using Multiple Energy Storage

Multiple energy storage can be used for demand-side energy management. A load hiding mechanism that obscures household consumption was proposed in [117]. In this model, the available household energy storage is used to reduce the cost of load hiding. To reduce the reliance on a RB for load hiding, it combines EV with HVAC systems. A Markov decision process was formulated to account for the stochastic nature of customer demand. The method uses a Q-learning algorithm to adapt the control policies for energy storage units. It formulates a deterministic optimization problem and derives its equivalent convex form to provide an idealized benchmark system. However, this method uses a large capacity RB, which can be costly for the consumers.

In this section, we discuss the techniques that mitigate threats from strong adversaries (by prioritizing cost-friendliness without sacrificing customers’ privacy). Table 7 summarizes the methods discussed in this section.

## 5. Challenges and Future Research Directions

In this section, we summarize the challenges in SM privacy-preservation methods presented in this survey paper, and provide potential future research directions in the field. We categorize these challenges and research directions into two categories, i.e., privacy-preserving techniques that aim to defend against (i) weak and (ii) strong adversaries.

### 5.1. Defending against Weak Adversaries

#### 5.1.1. Aggregation-Based Methods with TTP

The main challenge of aggregation-based methods with TTP is that a fully trusted third party has to be present. There is a possibility that the TTP may not be fully trusted. In some cases, it may act as an adversary or have contact with an adversary to gain benefits by sharing personal information of consumers. It also requires additional hardware for the TTP, which results in higher complexities in SMs and the infrastructure. Such approaches can be a technical and strategical burden. To this end, many approaches have proposed the use of privacy models without considering TTPs. The use of a decentralized TTP is a potential future research avenue. In a decentralized system, consumers interested in privacy will act as TTPs.

The information about one customer should be verified by other customers. With this policy, it is important to verify a consumer before adding the consumer to the TTP group. The privacy protection of one consumer depends on the trust of the other consumers.

TTPs can perform secure aggregation to confirm the privacy of SMs. However, these methods mainly focus on the privacy of SMs. It gives less priority to the utility of data. The SM complexity is lower in case of secure aggregation approaches with TTP. Most of the aggregation approaches with TTP partially use HE, resulting in higher complexities. A better future approach would allow the UP and consumers to identify if a TTP has compromised the privacy by using the SM data for any other purposes.

#### 5.1.2. Cryptography-Based Approaches

Cryptography-based approaches without TTP typically requires sophisticated computation software and infrastructure. In cryptography-based privacy-preserving billing, operations, and VAS, most of the approaches guarantee data confidentiality and integrity. However, they do not ensure resistance against sybil attack, non-repudiation, and auditability. Achieving this level of security can be very difficult while preserving the privacy of SMs. Future research should focus on achieving resistance against these attacks while preserving the privacy of SMs. Data tampering, key management, and utility are important issues to consider for efficient privacy preservation of SMs.

We can argue that aggregation approaches are critical in preserving the privacy of SMs. In aggregation-based schemes, consumers can be divided into groups. Each group aggregates their SM readings and send them to the UP. However, billing can be an issue. It is important to use high-resolution of individual SM data for highly accurate billing. The UPs have control over the transmission systems. They can embed the required sensors to know the energy requested by the houses. One of the exciting future directions can be aggregating the SM data by using the SM appliances used in common households. The UP can provide software that is compatible with the SM devices. Both billing accuracy and privacy preservation can then be ensured.

#### 5.1.3. Homomorphic Encryption (HE)

There are two types of HE, namely a partial homomorphic cryptosystem and a full homomorphic cryptosystem. A partially homomorphic system can perform either addition or multiplication whereas a full homomorphic system can perform both. Without any knowledge of the secret key, HE can perform computation on encrypted messages. The aggregator node is unable to read the actual information collected by the SM. Hence, HE schemes are secure against internal attacks.

They can overcome the problems with deterministic cryptosystems. Most of the schemes use HE to preserve privacy of SMs. However, HE schemes do not provide verifiable computing. SM data is sent to the aggregator node and the node compute some operations on the ciphertext. SM cannot verify the computation performed by the aggregator. Future research should focus on an additional mechanism to overcome this problem and enable the widespread use of elliptic curve cryptography for SM privacy.

#### 5.1.4. Random Noise-Based Privacy Mechanisms

The addition of random noise may affect the billing accuracy. The main challenge is determining the amount of noise that need to be added for an optimal trade-off between privacy and utility. An accuracy loss occurs for adding random noise to preserve the DP. There is a direct relationship between the induced noise power and the level of DP that the model can achieve. In some scenarios, the result may become lost in the noise and become useless for the utility and billing. DR-based techniques cannot properly function when random noise is added. The trade-offs between DP privacy and utility should be carefully considered and evaluated as part of future work.

#### 5.1.5. Data Management for Operations

One of the future research directions for privacy-preserving billing is the use of effective algorithms that operate the smart electric devices to preserve privacy. There is limited work in this area. For this mechanism to work, consumers need to be more co-operative. Rewards can be provided based on the cooperation of the consumers, and different trusted software programs can be used for accurate computation.

Since SM data are used in real-time for operations, it is necessary that the solution should have low computational overhead and can support distributed operations. The UP should manage and operate the SM data, and the entity itself (either UP or TTP) that manages the SM data should be fully trusted. Future research should investigate approaches that analyse the fundamental limitations of obfuscation-based approaches. Another possible solution is implementing prediction-based approaches where the predicted energy consumption for every TS are used for different operations.

#### 5.1.6. Blockchain-Based Methods

Blockchain exhibits a distributed framework in the form of public ledgers and miners. Limited studies have explored Blockchain-based methods for SM privacy. Blockchain-based methods typically incur higher power consumption, higher computation overhead, and scalability issues. Future research should focus on addressing these issues to make Blockchain more deployable in SMs.

Future research should also focus on privacy selling. Different energy consumers have different priorities for privacy. Some consumers and organizations may prioritize financial gains over privacy. Hence, the UP can offer monetary benefits for the consumers to share their energy consumption data by considering the variation for the priority of privacy. The monetary benefits may vary based on the level of sensitive information and the time of information shared. By adopting this approach, the UP will be able to accurately measure the amount of energy required to fulfil the demand of the consumers. The consumers who prioritize privacy are also able to have control over their level of privacy.

Privacy-preservation methods for RES-based households are in its early stage and have several limitations. Future research should overcome these limitations. For example, an RB can be used to hide the trace of the energy consumption from the RES to preserve the privacy of consumers. The RB can filter the amount of renewable energy added with the demand of the house. The RB can also store renewable energy and use it during the peak periods when the demand is high.

### 5.2. Defending against Strong Adversaries

#### 5.2.1. Constraints of Energy Storage Units

Energy storage has constraints, such as limited capacity and the maximum charge–discharge rate. For example, a RB has a maximum charge–discharge rate and limited capacity. There is also a limited capacity for energy management in solar panels, HVAC, and real/reactive power. The main challenge of managing demand-side energy is the use of limited capacity energy storage to preserve the privacy of SMs. However, this can be a challenge due to the constraints in energy storage. It is important to investigate the use of other available energy storage devices for energy management.

#### 5.2.2. Economic Privacy by Energy Storage

To defend against a strong adversary, consumers need to pay for an energy storage unit. There is maintenance cost and depreciation for the energy storage units. Cost-friendly privacy for demand-side energy management was proposed to overcome this issue. It is important to confirm that cost savings should be greater than or equal to the costs of using energy storage to preserve the privacy of SMs. Future directions should focus on comparing the costs of using an energy storage units for privacy with the cost savings by using the proposed model.

#### 5.2.3. Information Theory-Based Approaches

Information-theory-based approaches suffer from reproducibility of research results in the SG domain. Existing methods evaluate their performance by simulations using a binary load model. None of them use real SM data for performance evaluation. Future research should focus on using real SM data for simulations and aim to implement the model in the real-world.

#### 5.2.4. Differential Privacy

Due to the constraints of the energy storage (limited capacity and charge–discharge rate), these works consider the approximate DP. The main challenge for RB-based DP is the constraints of the energy storage. Approximate DP introduces an error to standard DP. By properly designing an EMU to generate random noise for approximate DP, it is possible to reduce the error introduced in approximate DP. Future research directions should focus on designing better approximate DP using limited capacity RB, RES, or both.

#### 5.2.5. Demand-Side Energy Management for DR

Future techniques could use both RB and RES for privacy-preserving DR of SMs. Renewable energy generated by the RES can be used to supply the consumption during the DR program. The extent of cost saving depends on the amount of demand reduction for a particular period for DR. By using the renewable energy stored in the RB, consumers can obtain the maximum energy reduction during the DR response period to achieve the maximum cost saving. It is possible to obtain cost-saving privacy for the DR program of the SM. To overcome the problems of SM data tampering, demand-side energy management using energy storage have already been proposed in the literature. However, demand-side energy management for DR is relatively new and could be explored further as part of future work.

#### 5.2.6. Use of Reactive Power with Energy Storage

SMs can measure reactive power with real power. Reactive power recorded in the SM can be used to infer the private information of a consumer [109]. The method in [109] considers reactive energy for SM privacy. It is important for future research to consider real and reactive power. We can consider energy management by using both real and reactive power to mask SM data. Energy management should also consider the cost reduction during the privacy preservation of SMs. It is essential to consider the management of reactive power by using reactive and capacitive devices to reduce the costs incurred in consumers households.

#### 5.2.7. Use of Repetitive Output Shape

From the existing methods reviewed in this paper, we observe that some approaches attempt to manage the energy storage device in such a way so that the output of the SM is always a constant value [28,29,31]. This can remove the trace of individual energy usage of electric appliances. Other methods attempt to make the output of the SM as a rectangle shape to overcome the trace of electricity appliances usage [114].

Future work can focus on using energy storage units to make the output of an SM as a pulse or a saw-tooth wave to remove the trace of electrical appliances from the output of the SM data. It is very difficult to make the output of the SM data to a constant value due to the constraints of the energy storage devices. However, it is comparatively easy to make the output of the SM data to a repetitive shape (such as a rectangular, pulse wave, or saw-tooth wave) by using limited capacity energy storage units. These outputs are variable, and small changes in these outputs are less observable compared to a constant output shape.

## 6. Conclusions

Recent developments in SG technologies have sparked extensive debates and discussions on SM privacy worldwide. A wide range of privacy-preservation techniques and algorithms have been proposed with emerging approaches becoming more complex and sophisticated. To this end, this paper reviewed the current status of SM privacy and extensively surveyed the state-of-the-art privacy-preserving techniques published in the literature over the past decade.

In this paper, we categorized these techniques based on the types of privacy threats, namely weak and strong adversaries, each with a varying degree of algorithmic and implementation complexity. Our review concluded that most techniques that aim to defend against weak adversaries are cryptography and aggregation-based methods. These privacy-preserving approaches typically involve altering SM data, which can be problematic in certain use cases. Techniques to defend against strong adversaries are primarily based on demand-side energy management techniques.

These techniques preserve customers’ privacy from the attackers, any third-parties involved, and the UP itself while also being immediately applicable to the existing SG infrastructure. In reality, the practicality of a particular privacy-preserving technique also depends heavily on the deployment costs, prompting our review on various cost-friendly privacy protection mechanisms. Finally, we summarized the current challenges in SM privacy and outlined potential future directions for the research community.

## Figures and Tables

**Table 1:** Comparison of survey papers published in smart meter privacy preservation.

Ref.	Privacy for				CFP	PM	TPC
	RES	Bill	VAS	Oper			
[8]	✗	✓	✗	✓	✗	✗	2008–2012
[9]	✗	✓	✗	✓	✗	✗	2008–2012
[10]	✗	✓	✗	✓	✗	✓	2010–2013
[11]	✗	✓	✗	✓	✗	✗	2007–2014
[12]	✗	✓	✗	✓	✓	✓	2010–2014
[13]	✗	✓	✓	✓	✗	✗	2008–2015
Our survey	✓	✓	✓	✓	✓	✓	2013–present

Oper: Operation, CFP: Cost-friendly privacy, PM: Privacy metric, and TPC: Time-line of papers considered.

**Table 7:** Summary of cost-friendly privacy-preservation techniques of SMs against strong adversaries (prioritizing cost-friendliness).

Ref.	Goals	Limitations	Metric	Energy Storage
[43,110]	Optimization between load variance and cost reduction using a heuristic algorithm	The total expected value of an RB charge/discharge must be zero, use of larger capacity RB	Variance, Electricity and battery operation cost	RB
[28,29]	Heuristic algorithm-based optimization between privacy and cost reduction	Lower privacy for the off-peak period	Variance, MI, Average energy cost	RB
[111]	Cost-friendly approximate DP (using Laplace distributed random noise) of SMs	Conditions of the probability density function not possible in practice	MI, CoS	RB
[112]	Information-theory-based optimal trade-off between privacy and cost reduction	Use of unrealistic load for performance evaluation	MI, CoS	RB
[113]	A trade-off between privacy and the expected cost of energy using unauthorized HT	Computationally inefficient	Kullback–Leibler divergence, CoS rate	RB
[114]	Hide both high-frequency and low-frequency consumption pattern using a machine-learning-based approach	Convergence time of reinforcement learning is long	MI, Correlation coefficient, Saving ratio	RB
[115]	Use weather prediction to preserve privacy and increase the efficiency of energy usage by heuristic algorithm-based approach	Use of high capacity RB, lack of cost-reduction analysis	Graphical comparison	RB, RES
[26]	Heuristic method-based cost-friendly privacy by model predictive controller	Use of high capacity RB, consider future consumer load is knowns	MI, Total energy cost	RB, RES
[116]	Information-theory-based privacy-energy efficiency trade-off in SM system	No use of real SM reading for simulation	MI	RB, RES
[117]	Obscures household consumption by using the heuristic algorithm based method	Use of a large capacity RB	MI, Expected total cost	Multiple energy storage

## Data Availability

Not applicable.

## Abbreviations

The following abbreviations are used in this manuscript:

AgG	Aggregation
AMI	Advanced metering infrastructure
AVISPA	Automated validation of internet security protocols and applications
BAN	Building area network
BLH	Battery-based load hiding
BP	Bilinear paring
CC	Control centre
CCO	Computation and communication overhead
CDH	Computational diffie-Hellman
CH	Cluster head
CO	Computation overhead
CoO	Communication overhead
CoS	Cost saving
CS	Charging station
DP	Differential privacy
DR	Demand response
EMU	Energy management unit
Enc	Encryption
ETD	Energy theft detection
EV	Electric vehicle
HAN	Home area network
HE	Homomorphic encryption
HT	Hypothesis testing
HVAC	Heating, ventilation, and air conditioning
LA	Local aggregator
MI	Mutual information
MPC	Model predictive controller
MRPL	Maximum reported peak load
NAN	Neighbouring area network
NILM	Non-intrusive load monitoring
NTRU	Number theory research unit
OLE	Onion-level encryption
PLC	Power line communication
PPN	Privacy-preserving nodes
RB	Rechargeable battery
Ref	Reference
RES	Renewable energy sources
RMSE	Root mean square error
ROR	Real-or-random model
RTU	Remote terminal unit
SD	Statistical distance
SDN	Software-defined network
SG	Smart grid
SM	Smart meter
SoC	State of charge
SQL	Structured query language
TS	Time slot
TTP	Trusted third party
UP	Utility provider
VAS	Value added services
VB	Vehicle battery
WAN	Wide area network
ZKP	Zero-knowledge proofs
